# Trace Metals Derived from Electronic Cigarette (ECIG) Generated Aerosol: Potential Problem of ECIG Devices That Contain Nickel

**DOI:** 10.3389/fphys.2016.00663

**Published:** 2017-01-10

**Authors:** Dominic L. Palazzolo, Andrew P. Crow, John M. Nelson, Robert A. Johnson

**Affiliations:** ^1^Department of Pharmacology and Physiology, DeBusk College of Osteopathic Medicine, Lincoln Memorial UniversityHarrogate, TN, USA; ^2^Department of Preclinical Sciences, College of Osteopathic Medicine, William Carey UniversityHattiesburg, MS, USA

**Keywords:** ECIG, E-liquid, vaping, smoking, aerosol, trace metals

## Abstract

**Introduction:** ECIGs are currently under scrutiny concerning their safety, particularly in reference to the impact ECIG liquids (E-liquids) have on human health. One concern is that aerosolized E-liquids contain trace metals that could become trapped in respiratory tissues and induce pathology.

**Methods:** To mimic this trapping, peristaltic pumps were used to generate and transport aerosol onto mixed cellulose ester (MCE) membranes where aluminum (Al), arsenic (As), cadmium (Cd), copper (Cu), iron (Fe), manganese (Mn), nickel (Ni), lead (Pb), and zinc (Zn) were subsequently captured and quantified. The presence of trace metals on unexposed MCE membranes and on MCE membranes exposed to mainstream smoke served as control and comparison, respectively. The presence of these metals was also determined from the E-liquid before aerosolization and untouched by the ECIG device. All metals were quantified using ICP-MS. The ECIG core assembly was analyzed using scanning electron microscopy with elemental analysis capability.

**Results:** The contents (μg) of Al, As, Cd, Cu, Fe, Mn, Ni, Pb, and Zn on control MCE membranes were 1.2 ± 0.2, 0.050 ± 0.002, 0.047 ± 0.003, 0.05 ± 0.01, 0.001 ± 0.001, 0.16 ± 0.04, 0.005 ± 0.003, 0.014 ± 0.006, and 0.09 ± 0.02, respectively. The contents of all trace metals on MCE membranes exposed to aerosol were similar to controls, except Ni which was significantly (*p* < 0.01) higher (0.024 ± 0.004 μg). In contrast, contents of Al, As, Fe, Mn, and Zn on MCE membranes exposed to smoke were significantly higher (*p* < 0.05) than controls. The contents of Al, As, Cu, Fe, and Mn on smoke-exposed MCE membranes were also significantly higher (*p* < 0.05) than their content on aerosol-exposed membranes. The contents *per* cigarette equivalent of metals in E-liquid before aerosolization were negligible compared to amounts of aerosolized E-liquid, except for Fe (0.002 μg before and 0.001 μg after). Elemental analysis of the core assembly reveals the presence of several of these trace metals, especially Al, Fe, Ni, and Zn.

**Conclusions:** In general, from the single ECIG-device/E-liquid combination used, the amount of trace metals from ECIG-generated aerosol are lower than in traditional mainstream smoke, Only Ni in the ECIG-generated aerosol was higher than control. The most probable source of Ni in this aerosol is the core assembly.

## Introduction

The use of electronic cigarettes (ECIG), referred to as “vaping,” has become extremely popular in American culture. Common reasons for their rise in popularity include ECIG use as an alternative to smoking and smoking cessation (Palazzolo, 2013). For many ECIG users, vaping is considered safer than smoking because tobacco is not burned; hence the thousands of toxic compounds associated with combustion of tobacco are not inhaled. But safer does not imply harmless and the question of ECIG safety is still under debate (Bhatnagar et al., 2014; Chapman, 2014; Oh and Kacker, 2014; Pisinger, 2014; Abrams and Niaura, 2015). In fact, there is much concern about the detrimental effects of ECIG-generated aerosol as perceived by the public, especially in the wake of two recent and highly publicized articles reporting hidden formaldehyde in ECIG-generated aerosols (Jensen et al., 2015) and DNA strand breaks and cell death induced by ECIG vapor (Yu et al., 2016). These articles claim that vaping is as dangerous as or more dangerous than traditional smoking without any substantial evidence to support their claims (Bates and Farsalinos, 2015; Holliday et al., 2016). On the other hand, evidence is also mounting showing there is an increase in dual use of ECIGs and conventional cigarettes (Filippidis et al., 2016; Kalkhoran and Glantz, 2016). The question of how this dual use might sway the balance from benefit to harm remains to be seen. It is worth noting that while the current evidence regarding ECIG safety is sparse, there are still no long term studies reporting severe health effects among ECIG users (Farsalinos et al., 2014; Hartmann-Boyce et al., 2016). Regardless of these concerns, there is still much that is not known about the effects and risks of ECIG use, particularly when it comes to inhalation of ECIG-generated aerosol.

Therefore, it is imperative that the physical characteristics and chemical composition of the inhaled aerosol be systematically investigated down to the nanoparticle level in order to determine the degree of safety. The challenges of such an undertaking are self-evident and complicated considering the sheer number of unregulated ECIG liquids (E-liquids) and the many types of ECIG devices that are available. Major considerations for the design of systematic experiments must include, but are not limited to, (1) how the ECIG-generated aerosol is to be collected for analysis so that a consistent methodology can be developed (i.e., the experimental design), (2) which brand of E-liquid and flavorings will be used in the study (i.e., commercially prepared or home brewed), (3) which components of the E-liquid are to be analyzed (i.e., metals, polycyclic aromatic hydrocarbons, etc.) and (4) which brand of ECIG device will be used in the study *(*i.e., different brands of ECIG devices are constructed of different materials). While comparing vaping to smoking might seem incommensurable, a reasonable attempt at comparison must be made in order to gauge the degree of safety of one inhalation behavior over the other, especially since vaping is deemed by many to be a safer alternative to smoking, despite the fact that nicotine is internalized in both behaviors. To illustrate this point, a recent study by Hahn et al. (2014), found nicotine, in a number of E-liquids, to be the only constituent of major concern.

As an original approach, a simple and effective methodology using peristaltic pumps and mixed cellulose ester (MCE) membranes to collect and trap ECIG-generated aerosol from a commercially available brand of E-liquid was first developed and validated. This system was then used to investigate the possibility that trace amounts of metals are present in the ECIG-generated aerosol at levels which could potentially impact respiratory tissues and induce pathology. This investigation reports the contents of aluminum (Al), arsenic (As), cadmium (Cd), copper (Cu), iron (Fe), manganese (Mn), nickel (Ni), lead (Pb), and zinc (Zn) recovered from MCE membranes after exposure to aerosol generated by a single device/refill fluid and compare them to the contents recovered after exposure to traditional mainstream smoke.

## Methods

### Puffing protocol

**Figure 1:**
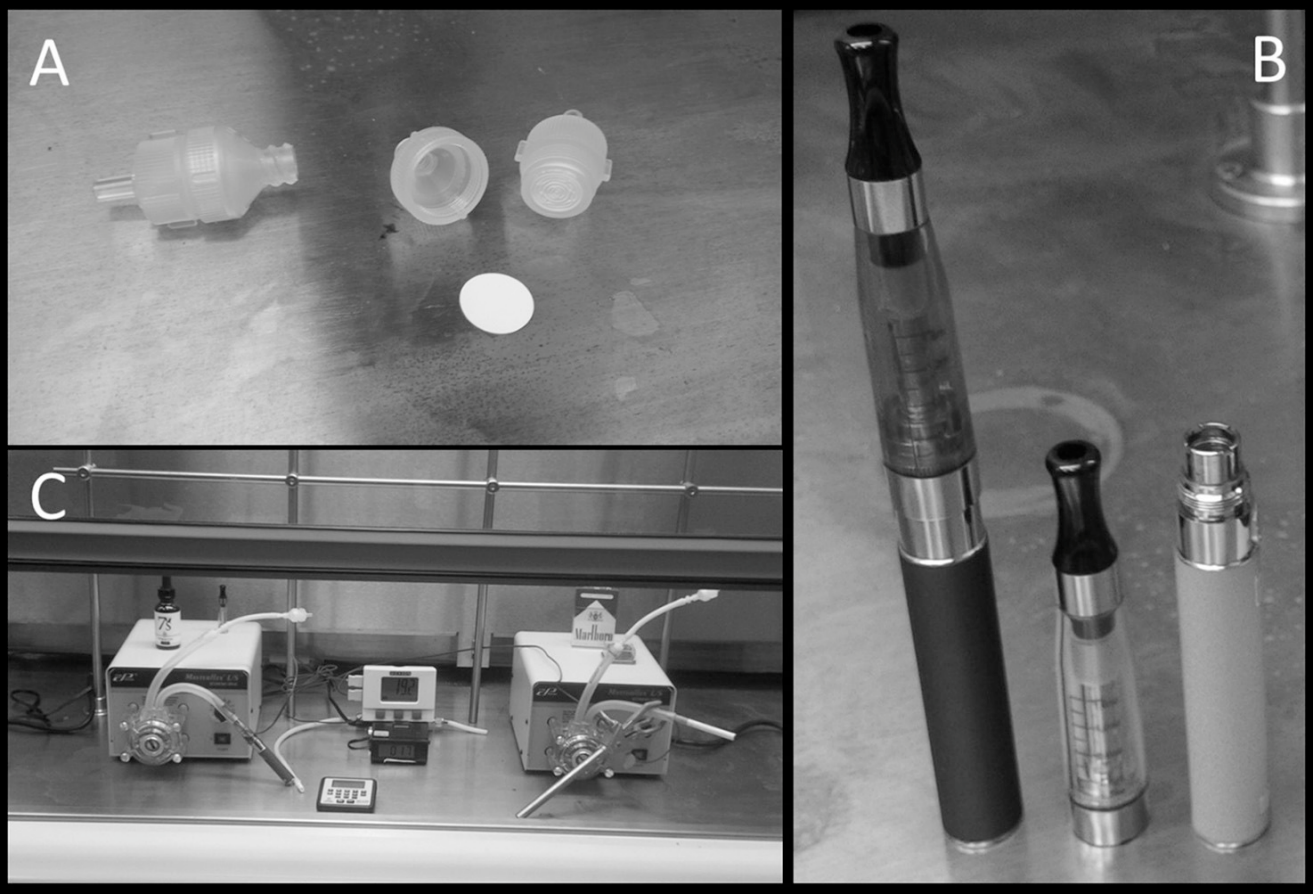
**Equipment used in the puffing protocol include (A)** Swinnex™ type filter holders and a Millipore Mixed Cellulose Ester (MCE) membrane, **(B)** Triple 3 eGo electronic cigarettes, and **(C)** peristaltic pumps in a Thermo Scientific Hamilton SafeAire II laminar flow hood.

Two Cole-Parmer MasterFlex L/S peristaltic pumps (Vernon Hills, IL) were used to simulate puffing on Triple 3 (Kennesaw, GA) eGo style ECIG devices or conventional Marlboro (84 mm, full strength) cigarettes. The ECIG devices vaporize 7 s, tobacco flavor, very high nicotine (South Lake, TX) brand of E-liquid. ECIG devices, E-liquid (in 15 or 30 ml bottles) and Marlboro cigarettes were all purchased from a local tobacco outlet. One peristaltic pump (the aerosol pump) was used to transport ECIG-generated aerosol through 12 inches of MasterFlex L/S 24 Precision Tubing (ID = 6.4 mm) onto a Millipore Mixed Cellulose Ester (MCE) membrane housed inside a Swinnex™ type filter holder (EMD Millipore Cooperation, Billerica, MA). A second peristaltic pump (the smoke pump) was used to transport smoke through an identical setup as the first peristaltic pump. The filter holders, which serve as in-line chambers, were perforated with a pin-prick sized hole in order to relieve excess pressure from the transported aerosol or smoke. The MCE membrane disks (13 mm diameter, 5 μm pore size, <5 mg dry weight) are made of mixed cellulose esters of acetates or nitrates containing less than 12.6% nitrogen (Figure 1A). To minimize cross contamination of pump tubing and in line chambers, the aerosol pump was used strictly for aerosol and the smoke pump strictly for smoke. Before each aerosol or smoke trial, pump flow rates were equilibrated to 400 ml/min using an Aalborg GFM flow meter (Orangeburg, NY) to simulate the flow of air intake during a 5 s puff. Filters were exposed to aerosol or smoke during 45 cycles of a 5 s puff (pump active) followed by a 10 s rest period (pump inactive), where 15 puffs approximates the extent of one cigarette. The Triple3 eGo devices, manufactured in China by JOMO Tech (2015), consist of a 650 mAh lithium ion battery (3.7 V, unregulated), a silicon ring at the base of the mouthpiece, and a plastic tank (i.e., “clearomizer”) with a 1.6 ml capacity to house the E-liquid. The resistance of the tank's heating coils varies between 2.2 and 2.6 Ω for an average power output of ~5.7 W (Figure 1B).

While details concerning the E-liquid specifications could not be obtained, conversations with representatives of the 7 s Electronic Cigarette company revealed that the E-liquid itself is a mixture of 80% propylene glycol and 20% vegetable glycerin (i.e., glycerol) containing 24 mg/ml of nicotine or ~3.4 mg nicotine/15 puffs. A trace of flavoring is added to the final E-liquid concoction to provide the tobacco taste. In comparison, a full strength Marlboro contains slightly less than 1.0 mg nicotine/cigarette (Calafat et al., 2004). All pump-puffing experiments were conducted within a Thermo Scientific Hamilton SafeAire II (Fisher Hamilton L.L.C., Two Rivers, WI) laminar flow hood equipped with a HEPA filter (Figure 1C). Laminar flow hood temperature and inlet and outlet temperatures of peristaltic tubing were monitored before and after each trial using a Dickson Temperature Logger (Addison, IL) equipped with dual flexible K-thermocouple temperature probes. To measure hood temperature, probe tips were left exposed inside the hood. To measure temperatures of inlet and outlet tubes, probe tips were placed ~1 cm inside the inlet or outlet tubes just before or after each trial.

### Anatomy of the core assembly

**Figure 2:**
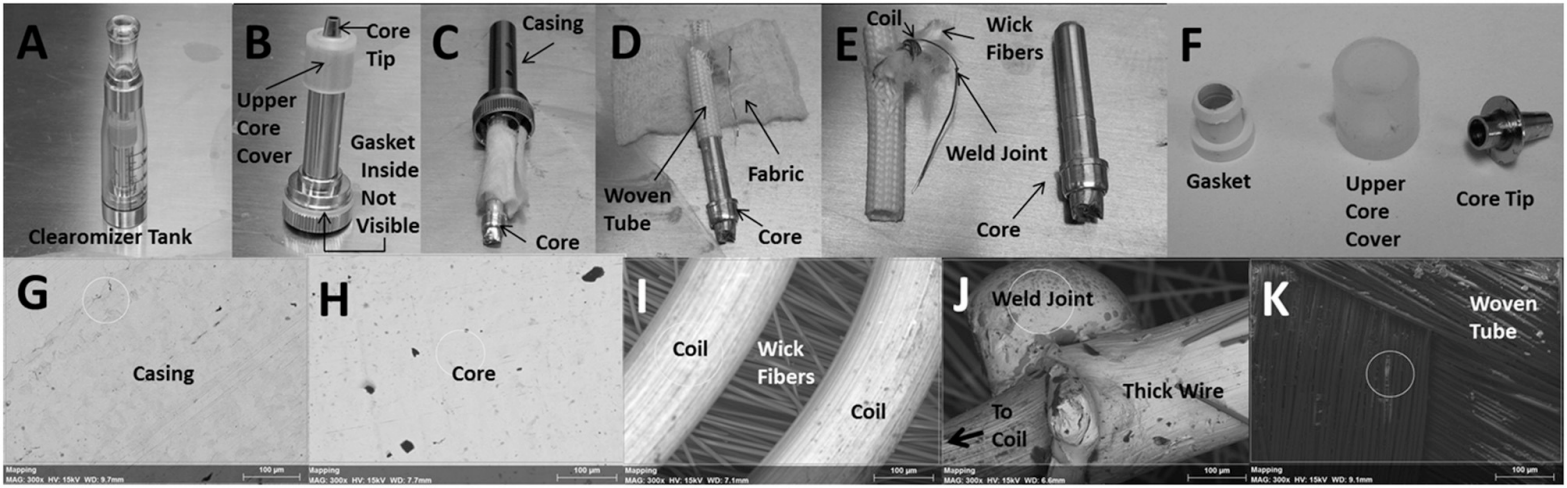
**Anatomy of the core assembly depicting (A)** plastic “clearomizer” tank, **(B)** encased core, **(C)** core wrapped in fabric, **(D)** core within woven tube, **(E)** exposed core, woven tube and coil and **(F)** gasket, upper core cover, and core tip. SEM images of the **(G)** inner surface of core casing, **(H)** core, **(I)** coils surrounding wick fibers, **(J)** weld joint between coil and extension wire and **(K)** inner surface of woven tube. The small white circle, where visible, indicates the area in which elemental analysis was performed (see Table 2). All SEM images were observed at an acceleration voltage of 15 kV and are depicted at a magnification of 300X.

The plastic tank contains the encased core as shown in Figure 2A. Figure 2B depicts the encased core with an upper core cover and core tip after it was removed from the plastic tank. Although not visible, inside the core casing is a gasket that helps secure the core within the casing. In Figure 2C, the core, wrapped with fabric material around a woven tube, was partially removed from the casing. Figure 2D shows the core after the fabric material was unwrapped and slipping out of the woven tube; notice also an exposed wire extending from inside the woven tube. In Figure 2E, the naked core is clearly visible and the resistance coil, which wraps around a clump of wick fibers, is fully exposed from within the woven tube; notice also the weld joint connecting the coil to the exposed wire from Figure 2D. The bottom of the core ultimately makes contact with the lithium ion battery. Figure 2F shows the gasket (after it was removed from inside the core casing), the upper core cover and the core tip. The following depictions are representative scanning electron microscope (SEM) images of the inner surface of the core casing (Figure 2G), the core (Figure 2H), the coils surrounding wick fibers (Figure 2I), the weld joint at the junction of the thin resistance coil and the thick extension wire (Figure 2J), and the inner surface of the woven tube (Figure 2K). The thick extension wire conducts the current from the bottom of the core to the resistance coil.

### Imaging of core assembly

The components within the core assembly of a brand new Triple 3 ECIG plastic tank, never exposed to E-liquid, were imaged using a Hitachi TM3000 (Hitachi, High-Technologies Corp, Dallas, TX) tabletop SEM equipped with a Bruker Quantax 70 (Bruker Optics, Billerica, MA) energy-dispersive X-ray spectrometer (EDS). The relative amounts of trace elements, as well as other elements with compositions greater than 5%, were determined. The presence of these trace metals on the core assembly were compared to their presence in E-liquid and to what was recovered from the MCE membranes following exposure to ECIG-generated aerosol. All SEM images of the core assembly were observed at an acceleration voltage of 15 kV and are depicted at a magnification of 300X.

### Imaging of MCE membranes

After MCE membranes were exposed to 0 (control), 5, 30, or 45 puffs of air, ECIG-generated aerosol, or smoke, the membranes were carefully removed from the inline chambers and mounted on 13 mm diameter aluminum stubs using 10 mm carbon impregnated double sided adhesive discs (Ladd Research Industries, Williston, VT). Microscopic images of the MCE membranes were obtained, and based on sampling area, the percentages of and the total numbers of carbon (C), oxygen (O), and nitrogen (N) atoms on each membrane were determined using the Hitachi TM3000 SEM equipped with a Bruker Quantax 70 EDS. All SEM images of MCE membranes were observed at an acceleration voltage of 15 kV and are depicted at a magnification of 3000X.

### Carbon monoxide analysis

Samples were immediately analyzed for carbon monoxide (CO) concentration from 100 ml (approximately 3 puffs) of air, aerosol, or smoke transported through the peristaltic pumps and determined, as previous described (Vreman et al., 2005; Johnson et al., 2006), via a customized solid phase gas chromatography unit (Peak Laboratories LLC, Mountain View CA). Briefly, quantification of CO involves the passing of gas samples through a heated mercury (Hg) column to release Hg vapor. This signal is, in turn, quantified via a photodiode and amplified to be compared with known CO standards. Using this well-established and highly selective method, CO levels can be accurately measured at 1.0 ± 0.5 ppb and higher. The rate of CO generation was calculated from pump outlet tube concentrations (in ppb) and flow rate (ml/min) at a point before the inline chamber. Hood air (control) was collected directly from inside the hood. All samples were collected using a 100 ml gas tight glass syringe of which 200 μl was manually injected into the gas chromatograph with the exception of the smoke samples which were first diluted 1000 fold before manual injection. Final concentrations of CO are presented in μM/L.

### Analysis of trace metal

The contents (μg) of Al, As, Cd, Cu, Fe, Mn, Ni, Pb, and Zn were determined from virgin MCE membranes (control; *n* = 9). The contents of these metals on MCE membranes were also determined after 45 puffs of ECIG-generated aerosol (*n* = 8) or cigarette smoke (*n* = 8). Additionally, the concentrations of these trace metals were determined in quadruplicate from one bottle of 7 s tobacco flavor, very high nicotine brand of E-liquid (i.e., before aerosolization and untouched by the ECIG device) and in triplicate from the tobacco and paper of three Marlboro cigarettes (686.7 ± 19.7 mg/cigarette, filter not included). All trace metal analyses were performed as a contracted service by the Environmental Health Sciences Laboratory of East Tennessee State University using a Bruker 820-MS (Bruker Daltonics Inc., Billerica, MA) inductively coupled plasma-mass spectrometry (ICP-MS). The E-liquid was first diluted in a 1% nitric acid to a final concentration of 1% E-liquid followed by ICP-MS analysis. The tobacco and paper of cigarettes and MCE membranes were subject to acid digestions according to the GFAA/ICP-MS digestion procedure outlined in Environmental Protection Agency protocol 3050B (United States Environmental Protection Agency, 1996). Certified “Trace Metals QC Standard (QCI-034-1),” manufactured by NSI Lab Solutions (Raleigh, NC) was used as a QC control for all cation analyses performed for this study. All QCs passed, with most in the 90–110% recovery range. ICP-MS analysis followed Environmental Protection Agency protocol 6020B (United States Environmental Protection Agency, 2014).

### Statistical analysis

With the exception of the percentages of trace metals determined by elemental analysis of the core assembly, all other values are presented as mean ± standard error (SE). Pearson's correlation coefficient (*r*) was used to determine if a linear relationship exists between the number of puffs on the ECIG device and the volume of E-liquid aerosolized where *r* > 0.700 indicates a strong positive correlation. Differences in temperatures (hood, inlet tubes, and outlet tubes) were determined using a two-way ANOVA followed by Tukey's *post-hoc* analysis to test for differences within and between treatment groups. Differences in the elemental compositions (C, O, and N) of MCE membranes and in the concentrations of trace metals recovered from MCE membranes were determined using a one-way ANOVA followed by Tukey's multiple comparison tests. For all comparisons, *p* < 0.05 indicated statistical significance.

## Results

### Validation of puffing protocol

**Figure 3:**
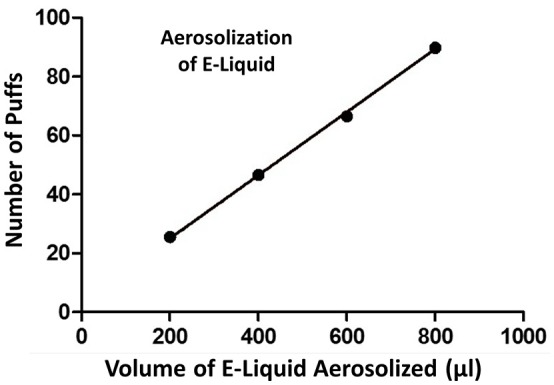
**Number of puffs on the ECIG device as a function of volume (μl) of E-liquid aerosolized**. Pearson *r* = 0.9995 and *p* < 0.005.

A plot of the number of puffs on the ECIG device as a function of volume of E-liquid aerosolized is shown in Figure 3. Each data point on the graph (i.e., XY pair) represents the average number of puffs (*n* = 3–5) to reduce the E-liquid volume in the plastic tank by 200, 400, 600, and 800 μl. From this data, a strong linear relationship is indicated and the amount of E-liquid aerosolized *per* 5 s puff is calculated to be 9.3μl or 419.9 μl/45 puffs.

The set flow rate for both the peristaltic pumps is consistently achieved from one experimental trial to the next. As indicated in Table 1, average flow rates for the aerosol (*n* = 24) and the smoke (*n* = 24) pumps are 402.7 ± 0.5 and 403.1 ± 0.4 ml/min, respectively. These flow rate results in a puff volume of about 33.6 ml *per* 5 s puff.

**Table 1:** **Percent recoveries of aerosol and smoke on MCE membranes**.

	**Aerosol pump**	**Smoke pump**
Flow rate	402.7 ± 0.5 ml/min (*n* = 24)	403.2 ± 0.4 ml/min (*n* = 24)
Puff duration	5 s	5 s
Puff volume	33.6 ml	33.6 ml
Weight of MCE membrane	4.45 ± 0.01 mg (*n* = 10)	4.45 ± 0.01 mg (*n* = 10)
Weight of E-liquid (Aerosol of 3 cigarettes equivalent) + MCE membrane	16.1 ± 0.2 mg (*n* = 10)	–
Weight of E-liquid on MCE membrane	11.7 mg	–
Weight of 420 μl of E-liquid (3 cigarettes equivalent)	448.4 mg	–
Percent recovery of aerosol	2 to 3%	–
Weight of particulate matter (Smoke of 3 cigarettes) + MCE membrane	–	9.9 ± 0.4 mg (*n* = 10)
Weight of particulate matter (Smoke of 3 cigarettes) on MCE membrane	–	5.4 mg
Weight of particulate matter *per* cigarette on MCE membrane	–	1.8 mg
Amount of particulate matter generated on MCE membrane	–	0.01 mg/ml/s
Percent Recovery of Smoke^*^	–	5 to 6%

* *Percent recovery of smoke is based on Calafat et al. (2004) value of 13.4 mg of tar per Marlboro cigarette and Thielen et al. (2008) value of 4.5% of smoke as particulate matter. Tar is essentially the same as particulate matter, minus the water content (see discussion). Values given as mean ± SEM*.

The percent recoveries of aerosol and smoke after 45 puffs are also shown in Table 1. The weights of the MCE membranes before and after aerosolization (45 puffs) are 4.5 ± 0.8 and 16.1 ± 0.2 mg, respectively, resulting in a weight of 11.7 mg of E-liquid on the MCE membrane and the percent recovery of E-liquid on the MCE membrane is between 2 and 3% after 45 puffs of the ECIG device. The average weight of an MCE membrane exposed to 45 puffs of smoke is 9.9 ± 0.4 mg, resulting in a weight of 5.4 mg of particulate matter on the MCE membrane after 45 puffs. The percent recovery of smoke on the MCE membranes is between 5 and 6%.

**Figure 4:**
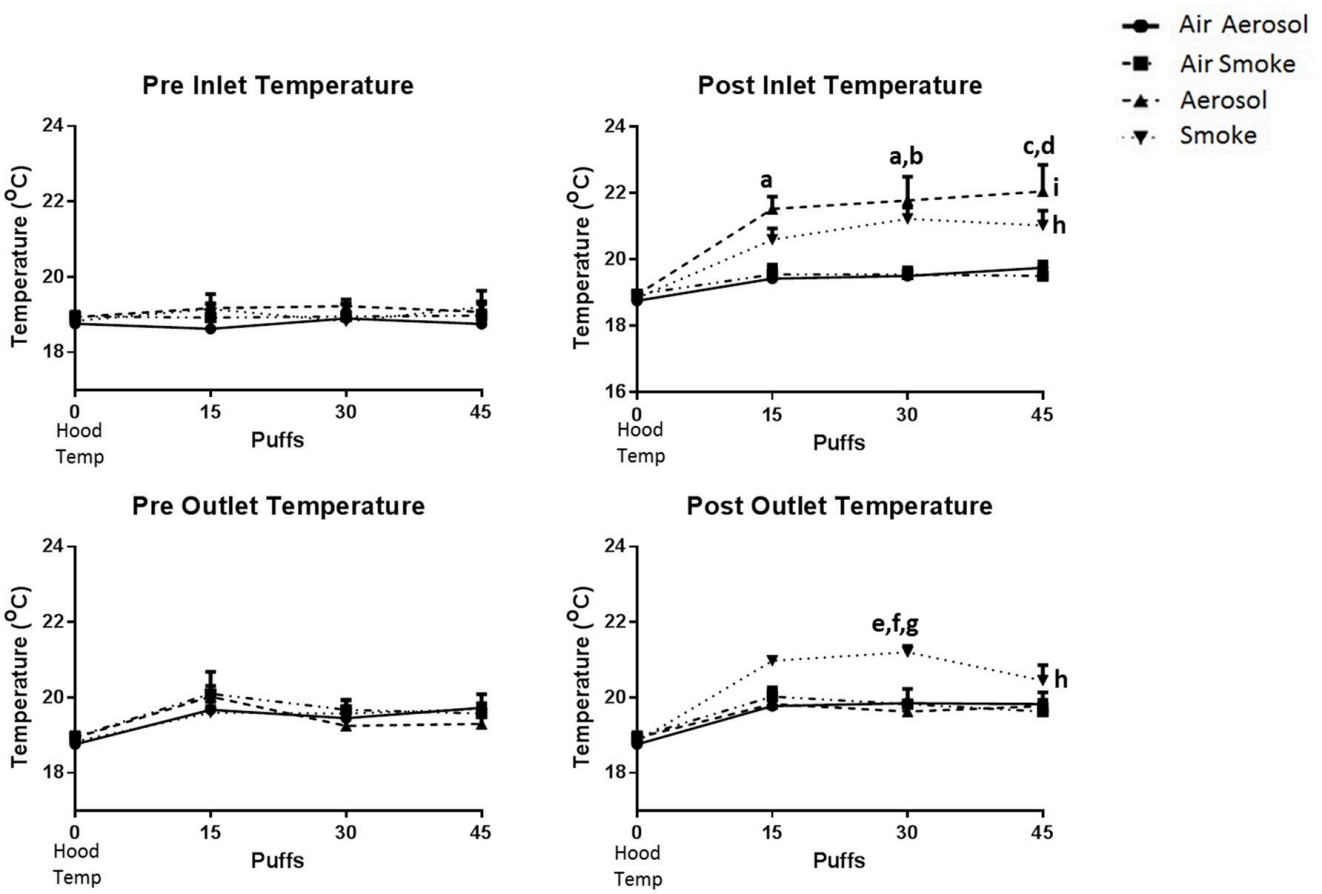
**Temperatures within the laminar flow hood and within the inlet and outlet peristaltic pump tubing, before and after pumping air, ECIG-generated aerosol, and smoke**. Data points given as mean ± standard error of the mean. Post-inlet between group comparisons; a = *p* < 0.05 at 15 and 30 puffs between aerosol and air through the aerosol pump, b = *p* < 0.05 at 30 puffs between aerosol and air through the smoke pump, c = *p* < 0.01 at 45 puffs between aerosol and air through the aerosol pump, and d = *p* < 0.01 at 45 puffs between aerosol and air through the smoke pump. Post-outlet between group comparisons at 30 puffs; e = *p* < 0.05 between smoke and air through the aerosol pump, f = *p* < 0.05 between smoke and air through the smoke pump and g = *p* < 0.01 between smoke and aerosol. Within group comparisons between hood temperature (control) and exposure to smoke (h = *p* < 0.05) or aerosol (i = *p* < 0.05).

The temperatures within the laminar flow hood (control, *n* = 3) and within the inlet and outlet peristaltic pump tubing (*n* = 4), both before and after pumping air, ECIG-generated aerosol and smoke is depicted in Figure 4. Hood temperatures (shown as 0 puffs) range between 18.6 and 19.2°C. Comparisons made between groups (i.e., air through aerosol pump, air through smoke pump, ECIG-generated aerosol, and smoke) indicate there is no statistical difference in the temperatures of both pre-inlet and pre-outlet tubes at 15, 30, or 45 puffs. Similarly, for air through the aerosol pump and air through the smoke pump, there is no difference in temperatures from both post-inlet and post-outlet tubes at 15, 30, or 45 puffs. In contrast, post-inlet temperatures for ECIG-generated aerosol (but not smoke) is higher than for air transported through the aerosol or smoke pumps at 15, 30, and 45 puffs. Conversely, post-outlet temperatures for smoke are higher than for air transported through aerosol or smoke pumps and aerosol pumped through the aerosol pump only at 30 puffs. Comparisons made within groups, indicate there is no relevant variance in pre-inlet and pre-outlet temperatures when compared to control hood temperature. Similarly, no variance is noted within groups when comparing hood temperature with temperatures in the inlet and outlet tubes after (i.e., post-) pumping air. Post-inlet temperatures for ECIG-generated aerosol and smoke both increase above hood temperature, but only post-outlet temperatures for smoke increases above hood temperatures.

### SEM analysis of core assemblies

The relative amounts of nine trace metals and four other key elements are given in Table 2 as a percentage of the various parts of the core assembly. All parts of the core assembly analyzed come in contact with the E-liquid and the values given represent the typical elemental compositions determined from several core assemblies. The core casing, both inner and outer surfaces, is comprised primarily of Fe (between 78 and 83%) and some Mn (between 13 and 16%). The core tip is made up primarily of Ni (81%) with some Cu (13%), and Zn (5%). The upper core cover is of rubbery consistency and comprised of 85% silicon (Si). The gasket contains Zn (41%), Si (32%), and Pb (16%). The fabric material consists of high percentages of Cu (43%) and Ni (24%). The woven tube, both outer and inner surfaces, is comprised primarily of Si (between 52 and 59%), tin (Sn; between 13 and 17%) and some Al (between 9 and 10%. The core itself, both upper and lower halves, appears to be coated with more than 72% silver (Ag) with underlying metal compositions of Ni (between 13 and 18%) and Cu (between 5 and 7%). The wick fibers within the surrounding resistance coil consist almost entirely of Si (87%). The coil filament around the wick fibers is high in Ni (76%) with less amounts of Si (9%) and Mn (9%). Similarly, the weld joint connecting the coil with the thick extension wire is made up of high amounts of Ni (84%) and some Si (9%). The thick extension wire beyond the weld joint is made up of mostly Ni (89%) with a minimal amount of Cu (7%). The juncture of thick extension wire, coil and weld joint contains 53% Ni and is the only place in the core assembly where levels of chromium (Cr; 18%) exceeds the 5% threshold.

**Table 2:** **Elemental analysis of the core assembly using EDS**.

**Core assembly**	**Trace metals**									**Other key elements**			
	**Al**	**As**	**Cd**	**Cu**	**Fe**	**Mn**	**Ni**	**Pb**	**Zn**	**Ag**	**Cr**	**Si**	**Sn**
Core casing (Outer surface)					83^*^	13							
Core casing (Inner surface)					**78**	16							
Core tip				13			**81**		5				
Upper core cover							7					**85**	
Gasket								16	**41**			32	
Fabric material^@^				**43**			24	5				8	
Woven Tube (Outer surface)^#^	9											**59**	13
Woven tube (Inner surface)^#^	10	5										**52**	17
Core (Bottom half)				7			13			**75**			
Core (Top half)				5			18			**72**			
Wick fibers (Within the surrounding coil)							8					**87**	
Coil (Around wick fibers)						9	**76**					9	
Weld joint							**84**					9	
Thick wire beyond weld joint				7			**89**						
Juncture of thick wire, coil, and weld joint							**53**				18		

* *Values are given as a weight percentage. Only values exceeding a 5% threshold are recorded in the table. The value of the element with the greatest percentage for each part of the core assembly is indicated in bold*.

@ *Presence of gallium (7%) may be a possible misidentification of Zn*.

# *Presence of Antimony (12%) may be a possible misidentification of Sn*.

### Visual inspection and SEM analysis of MCE membranes

**Figure 5:**
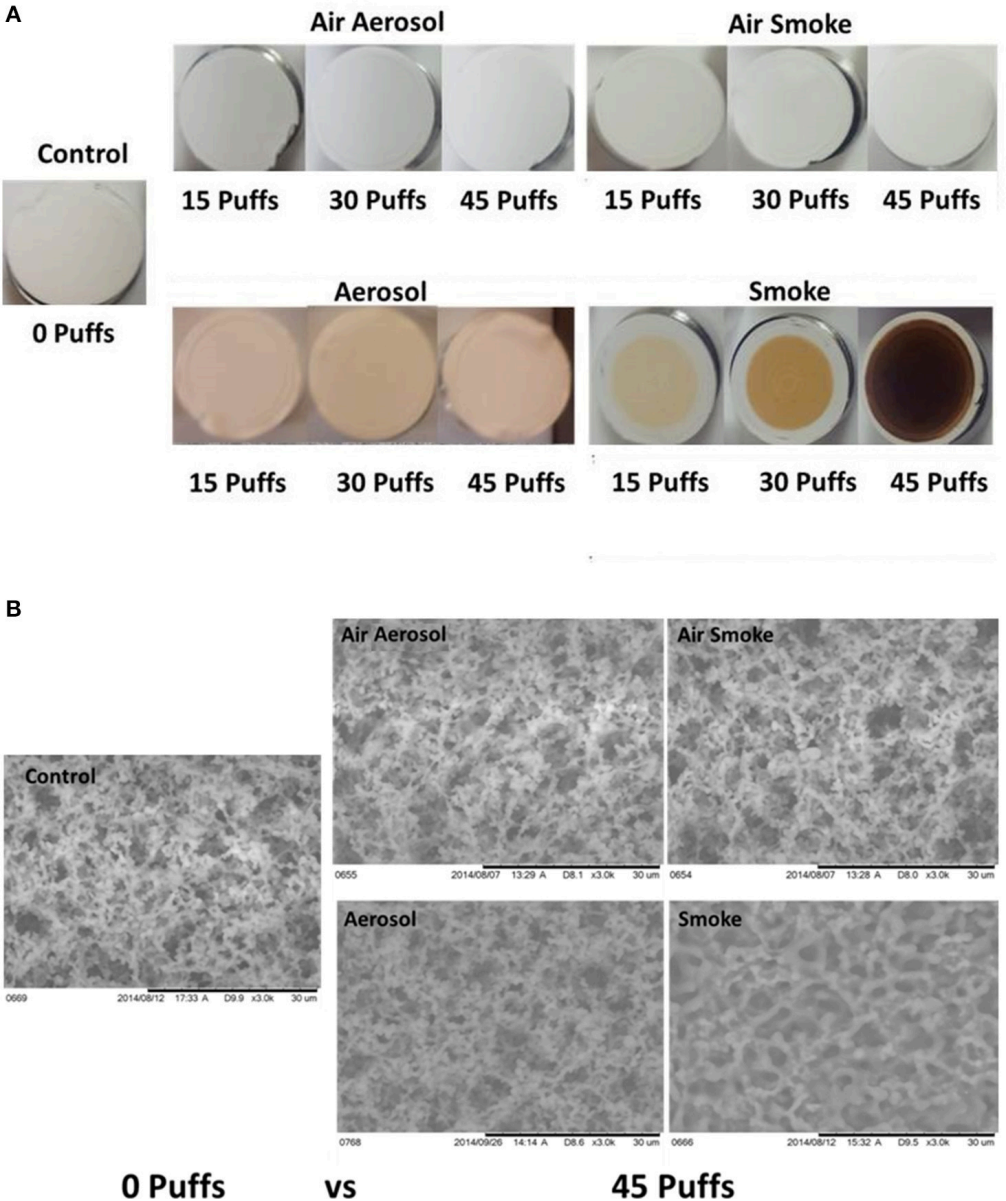
**(A)** Visual inspection Of MCE membranes after 0, 15, and 45 puffs of air, ECIG-generated aerosol and smoke and **(B)** SEM analysis of MCE membranes after exposure to 0 and 45 puffs of air, ECIG-generated aerosol and smoke. All SEM images shown at 3000X.

Results of visual inspection and SEM analysis of MCE membranes are shown in Figures 5A,B, respectively. Visual appearance and SEM images of membranes exposed to 15, 30, or 45 puffs of air through either the aerosol or smoke pumps appear the same as the control virgin MCE membranes. In contrast, 15, 30, and 45 puffs of ECIG-generated aerosol saturate and stain the membranes with E-liquid, giving the membranes a pinkish appearance consistent with the color of the E-liquid. No other conspicuous visual or SEM differences are observed. Exposures to 15, 30, and 45 puffs of smoke stain the membranes in an increasing color gradient ranging from light beige to dark brown. SEM images of these same MCE membranes revealed thicker membrane fibers and loss of fiber detail after 45 puffs of smoke.

**Figure 6:**
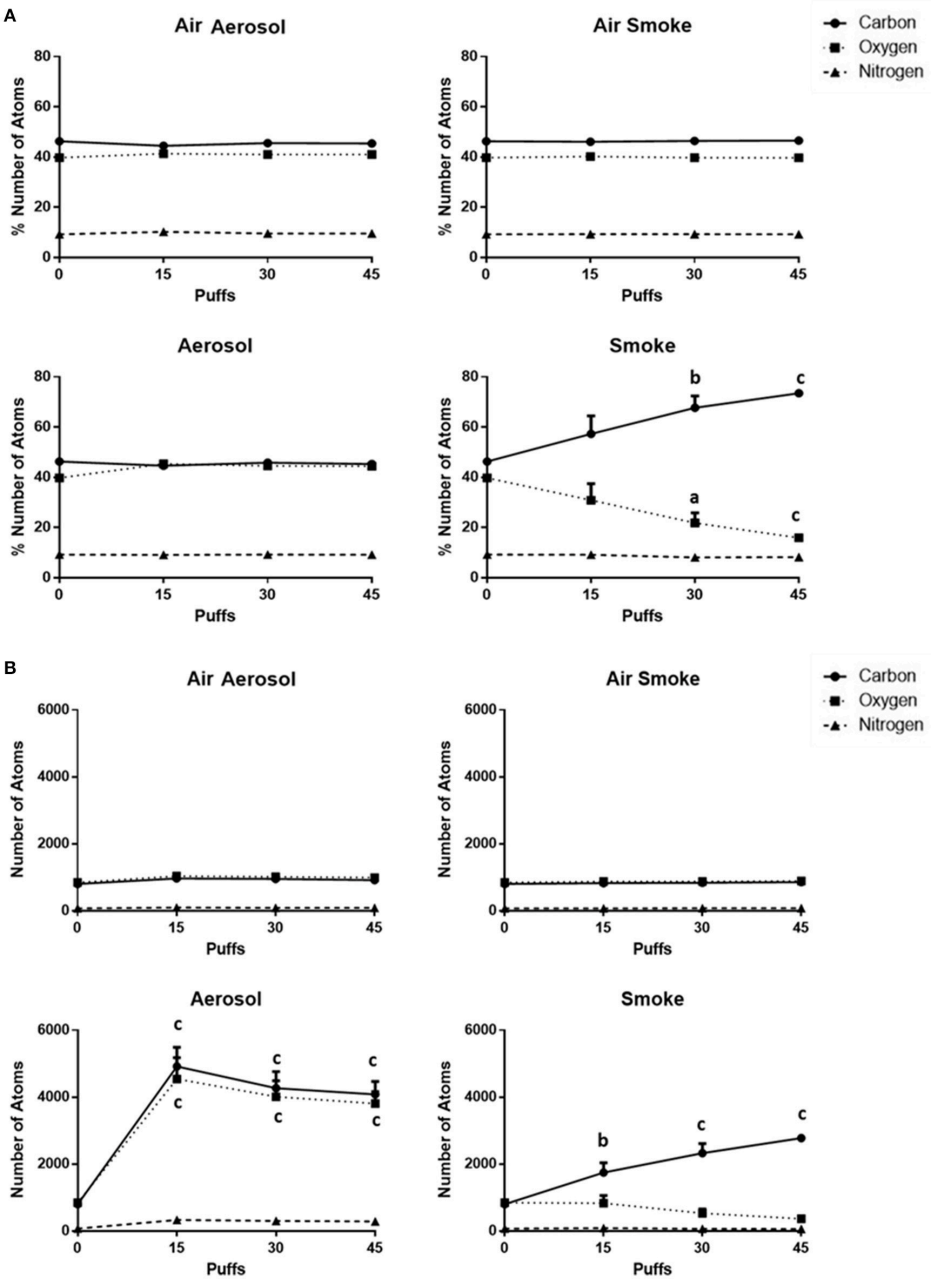
**(A)** Percentages of and **(B)** total numbers of (based on sampling area) C, O, and N atoms on MCE membranes after exposure to 0, 15, 30, and 45 puffs of air, ECIG-generated aerosol and smoke. Data points given as mean ± standard error of the mean. Comparisons between 0 puffs (control) and 15, 30, or 45 puffs where a = *p* < 0.01, b = *p* < 0.005 and c = *p* < 0.001.

The percentages of and total number counts of C, O, and N atoms on MCE membranes (*n* = 4) are shown in Figures 6A,B, respectively. Average percentages of C (range of 44.5–46.6%), O (range of 39.7–45.4%), and N (range of 9.1–10.3%) exposed to air through the aerosol and smoke pumps, as well as for ECIG-generated aerosol, remain constant regardless of number of puffs. In contrast, after exposures to 15, 30, and 45 puffs of smoke, the average percentage of C gradually increases from 46.3 ± 0.3 to 73.4 ± 0.5% while the average percentage of O gradually decreases from 39.8 ± 0.3 to 15.9 ± 0.3%. The average percentage of N remains constant between 9.2 ± 0.2 and 8.2 ± 0.3%. The average number count for C (range of 801–969), O (range of 847–1039), and N (range of 72–98) exposed to air through the aerosol and smoke pumps remain constant regardless of number of puffs. In contrast, after exposures of 15, 30, and 45 puffs of ECIG-generated aerosol, the average number of C atoms increases to 4918 ± 568, 4266 ± 496, and 4081 ± 384, respectively, and the average number of O atoms increases to 4540 ± 638, 4014 ± 472, and 3807 ± 354, respectively. While the average number of N atoms increases slightly after exposure to 15, 30, and 45 puffs of smoke, this increase is not significant. After exposures of 15, 30, and 45 puffs of smoke the average number of C atoms increases to 1746 ± 291, 2328 ± 283, and 2776 ± 61, respectively and the average number of O atoms decreases to 835 ± 230, 531 ± 129, and 366 ± 17, respectively, but this decrease did not achieve significance. The average number of N atoms remains constant after exposure to 15, 30, and 45 puffs of smoke.

### Analysis of carbon monoxide

**Figure 7:**
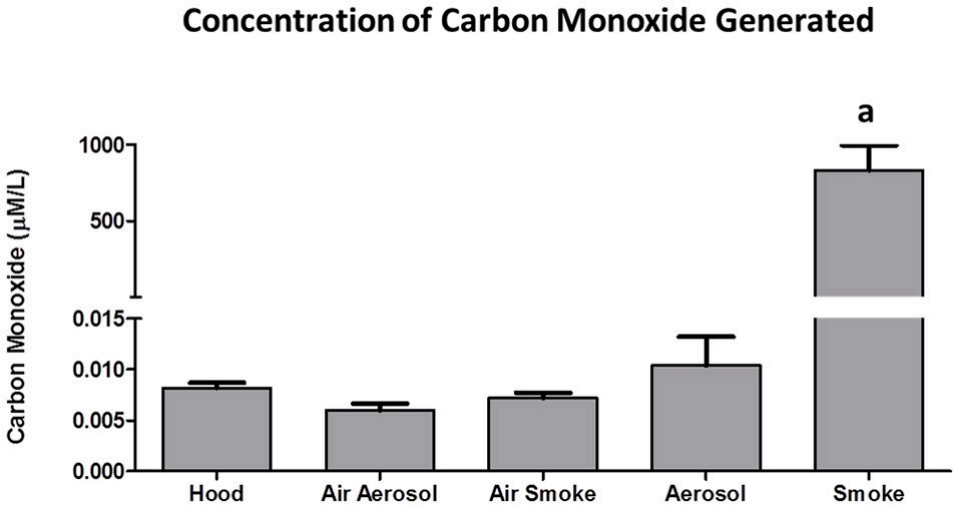
**Concentrations of CO collected from 3 puffs of air, ECIG-generated aerosol, and smoke**. Data points given as mean ± standard error of the mean. a = *p* < 0.001.

The concentrations of CO (*n* = 5) collected from 3 puffs of air, aerosol, and smoke are shown in Figure 7. Air in the hood, or transported through the aerosol and smoke pumps, as well as ECIG-generated aerosol, produced CO concentrations that range between 0.006 ± 0.001 and 0.010 ± 0.003 μM/L. In contrast, smoke generates an average CO concentration of 831 ± 166 μM/L.

### Analysis of trace metals

The concentrations of Al, As, Cd, Cu, Fe, Mn, Ni, Pb, and Zn in E-liquid (μg/L) and in the tobacco and paper of Marlboro cigarettes (μg/g) along with their contents (μg) based on 15 puffs (140 μL of E-liquid) of the ECIG device or 15 puffs of a cigarette (0.687 g of tobacco and paper) are listed in Table 3. With the exception of As, these results indicate that the content of all trace metals, on a *per* cigarette basis, are at least an order of magnitude higher in the tobacco and paper of a cigarette as compared to the E-liquid. Although, the content of As in the E-liquid is quite low, the As in the tobacco and paper is below the detection limit.

**Table 3:** **Trace metals in E-liquid before aerosolization and cigarettes before combustion**.

**Metal Analyzed**	**Al**	**As**	**Cd**	**Cu**	**Fe**	**Mn**	**Ni**	**Pb**	**Zn**
Concentration in E-liquid (μg /L) Mean^*^ ± SE	7.7 ± 0.5	0.08 ± 0.04	BDL < 0.01 μg/L	BDL < 0.01 μg/L	4.1 ± 0.2	0.159 ± 0.006	0.161 ± 0.007	BDL < 0.01 μg/L	0.51 ± 0.03
*n*	4	4			4	4	4		4
Content in E-liquid^@^ (μg/cig equivalent)	0.0032 ± 0.0002	0 ± 0	BDL	BDL	0.0017 ± 0.0001	0 ± 0	0 ± 0	BDL	0 ± 0
*n*	4	4			4	4	4		4
Concentration in tobacco (μg/g) Mean^#^ ± SE	348.8 ± 1.3	BDL < 0.1 μg/g	0.34 ± 0.03	6.3 ± 0.2	354 ± 4	105.4 ± 0.9	2.13 ± 0.01	0.77 ± 0.08	17.5 ± 0.6
*n*	3		3	3	3	3	3	3	3
Content in cigarette^!^ (μg/cig)	239.5 ± 0.8	BDL	0.26 ± 0.02	4.3 ± 0.2	243 ± 3	72.4 ± 0.6	1.467 ± 0.009	0.53 ± 0.06	12.0 ± 0.4
*n*	3		3	3	3	3	3	3	3

* *Determined (in quadruplicate) from one bottle of 7 s tobacco flavor, very high nicotine brand of E-liquid*.

@ *One cigarette is equivalent to 140 μl of E-Liquid*.

# *Determined (in triplicate) from the tobacco and paper (not including filter) of full flavor Marlboro cigarettes*.

! *Each cigarette is equivalent to 0.687 g*.

*BDL = below detection limit and are 0.01 μg /L for al trace metals in E-liquid and 0.1 μg /g for all trace metals in tobacco and paper*.

The content (μg) of all trace elements on control MCE membranes and on MCE membranes exposed to 45 puffs of ECIG-generated aerosol and conventional cigarette smoke are listed in Table 4. One value for Fe and five values for Ni on MCE membrane exposed to smoke were unrealistically higher than their upper detection limit (>130 and >1389 μg/45 puffs, respectively) and were not used for statistical evaluations. One value for Ni on the control MCE membranes is below the detection limit (<0.0005 μg/45 puffs) and was similarly not used. None of the analyzed trace metals on aerosol-exposed MCE membranes are significantly different from control membranes, except for Ni, which is nearly five times higher than the Ni content on control membranes. The contents of Al, As, Fe, Mn, and Zn on MCE membranes exposed to smoke are significantly higher than on control membranes. Similarly, the contents of Al, As, Cu, Fe, and Mn on MCE membranes exposed to smoke are significantly higher than those found on membranes exposed to aerosol. Since five out of the original eight Ni samples unrealistically exceeded the upper detection limit, any statistical evaluation using the results of Ni (with *n* = 3) trapped on MCE membranes exposed to smoke would lack confidence.

**Table 4:** **Contents of biologically active trace metals captured on MCE membranes**.

**Metal Analyzed**	**Al**	**As**	**Cd**	**Cu**	**Fe**	**Mn**	**Ni**	**Pb**	**Zn**
Control mean^*^ ± SE	1.2 ± 0.2	0.050 ± 0.002	0.047 ± 0.003	0.05 ± 0.01	0.001 ± 0.001	0.16 ± 0.04	0.005 ± 0.003	0.014 ± 0.006	0.09 ± 0.02
*n*	9	9	9	9	9	9	7	9	9
Aerosol mean^@^ ± SE	1.6 ± 0.3	0.050 ± 0.003	0.044 ± 0.003	0.019 ± 0.003	0.0011 ± 0.0002	0.13 ± 0.01	0.024^**e**^ ± 0.004	0.006 ± 0.002	0.17 ± 0.07
*n*	8	8	8	8	8	8	8	8	8
Smoke mean^#^ ± SE	2.7^**b, d**^ ± 0.2	0.059^**a, c**^ ± 0.002	0.062 ± 0.008	0.06^**c**^ ± 0.01	0.005^**a, c**^ ± 0.002	0.5^**a, c**^ ± 0.2	>UDL	0.017 ± 0.005	0.3^**a**^ ± 0.1
*n*	8	8	8	8	7	8		8	8

* *Content given as μg of biological trace metal on MCE membrane*,

@ *content given as μg of biological trace metal on MCE membrane exposed to 45 puff of aerosol*,

# *content given as μg of biological trace metal on MCE membrane exposed to 45 puff of smoke. UDL = above detection limit (>1389 μg/45 puffs)*.

*a = p < 0.05 for smoke vs. control; b = p < 0.005 for smoke vs. control; c = p < 0.05 for smoke vs. aerosol; d = p < 0.01 for smoke vs. aerosol; e = p < 0.01 for aerosol vs. control*.

## Discussion

In this study a smoke/aerosol system which can be used to effectively measure trace metals (as well as other low concentration compounds) in conventional cigarette smoke or ECIG-generated aerosol was established. In addition, a number of characteristics of ECIG vapors that differ from traditional cigarette smoke have been identified.

It is understood there is extreme variability in puffing topography between individuals who use ECIGs. This is also the case among smokers. When this work was started, very few studies were available (for both machine vaping and human vaping) to indicate a consistent set of parameters which should be used in ECIG research. This was further complicated when trying to establish a consistent set of parameters that would work for both vaping and smoking behaviors in a single study. In an investigation by Goniewicz et al. (2013a), a puff volume of 70 ± 68 ml and a puff duration of 1.8 ± 0.9 s was determined from eight male ECIG users, giving an estimated aerosol flow rate of 2333 ml/min during the puff. Their interpuff duration was 10 ± 13 s and they estimated that 15 puffs on an ECIG device is equivalent to one conventional cigarette. Assuming that 15 puffs on an ECIG device is equivalent to one cigarette; it was determined that a puff of 5 s duration with a pump flow rate of 400 ml/min and a puff volume of 33.6 ml was enough to finish a Marlboro cigarette almost to the butt. According to Zacny and Stitzer (1996), using data from more than 30 reports, the number of puffs/cigarette ranged from 8 to 16, the interpuff interval ranges from 18 to 64 s, the puff duration ranges from 1.0 to 2.4 s and the puff volume ranges from 21 to 66 ml. In this investigation, puff number and puff volume fall within these ranges. However, longer puff duration was chosen in exchange for lower flow rate so as to not damage the fragile MCE membranes. An interpuff duration of 10 s was selected to ensure the ECIG device did not shut down due to overheating, while keeping the length of time it takes to go through the smoking or vaping trials at a minimum. Until there is a concerted effort among all researchers in the ECIG research arena to make puffing topography (using puffing machines) uniform, these puffing parameters will continue to be used so as to maintain consistency in the data generated by our laboratory.

The percent recovery of aerosol on the MCE membranes is easy to determine given that the weight of the E-liquid (3 cigarettes equivalent) aerosolized onto the MCE membrane and the weight of 420 μl of E-liquid (3 cigarettes equivalent) before aerosolization are given (see Table 1). The percent recovery of smoke on the MCE membranes is harder to ascertain. However, Calafat et al. (2004) determined that the amount of tar produced from one Marlboro cigarette is 13.4 mg, which represents about 4.5% of total smoke (Thielen et al., 2008). If one accounts for the differences in the number of cigarettes smoked (1 cigarette for Calafat et al.; 3 cigarettes in this investigation), puff volume (35.0 ml for Calafat et al.; 33.6 ml in this investigation), and puff duration (2 s for Calafat et al.; 5 s in this investigation), and if it is assumed that nearly all the particulate matter is tar (Thielen et al., 2008), it is calculated that between 5 and 6% of the smoke is recovered on the MCE membranes.

A curious finding of the aerosol/smoke puffing system is the temperature difference between inlet and outlet tubing after vaping and smoking. Generally, the temperature of a burning cigarette is hotter than the temperature of vaporized E-liquid. Combustion of a cigarette generally produces temperatures that are greater than 800°C (Thielen et al., 2008). Although, the vaporization of propylene glycol based E-liquids depends on the voltage and resistance of the coil inside the tanks, theoretical vaporization temperatures have been estimated to reach as high as 350°C (Kosmider et al., 2014). While ECIG-generated aerosol and smoke temperatures are both higher than hood temperature in the inlet tube, the aerosol temperature is greater than smoke temperature. On the other hand, the smoke temperature in the outlet tube remains higher than hood temperature while aerosol temperature returns to hood temperature. A likely reason for this observation is differences between the physical natures of the aerosol (which is made up of liquid droplets) and the smoke (which is made up mostly of gas and particulate matter). In the inlet tube, it is possible that the drop in temperature of smoke (from its combustion point) is greater than the drop in temperature of the aerosol (from its vaporization point) because the liquid nature of the aerosol allows it to retain heat for a longer period of time. However, this does not explain why the temperature of the aerosol is lower than the temperature of the smoke in the outlet tube. It is possible that as the aerosol travels from inlet to outlet, contact with the inner wall of the pump tubing contributes to the more rapid decline in temperature and would also help explain why the percent recovery of aerosol on MCE membranes is less than percent recovery of smoke. Albeit the percent recoveries of both aerosol and smoke are both low, the puffing protocol used in this study is still effective in trapping elemental components onto the MCE membranes for the purpose of detecting differences in the delivered constituents.

EDS analysis reveals the percentages of C, O, and N of unexposed control MCE membrane are approximately 46, 40, and 9%, respectively. These values are close to expected for filters made of mixed cellulose esters (of acetate and nitrate) claiming less than 12.6% nitrogen (Millipore Safety Data, 2011). When MCE membranes are exposed to air or aerosol, percentage values of these elements remain close to the percentage values of the controls, regardless of number of puffs. In contrast, smoke gradually increases the percentage of C to 73% and gradually decreases the percentage of O to 16% while the percentage of N remains close to control. The reason for this increase in the C to O ratio is likely due to the particulate phase of whole cigarette smoke (Thielen et al., 2008) that layers the top of the filter, thus altering the composition of C, O, and N visible to EDS analysis. While smoke increases the C to O ratio, it is the aerosol that deposits more total atoms to the MCE membrane. This is most likely due to the liquid nature of the aerosol in comparison to the gaseous nature of the smoke. Despite the increase in the total number of atoms deposited by the ECIG-generated aerosol, the percentages of C, O and N remain constant, reflecting similarity in the percentages of C, O, and N between the E-liquid and the MCE membrane.

According to a 2012 report by the EPA (United States Environmental Protection Agency, 2012), the national standard for CO is not to exceed 9 ppm (or 236 μM/L) on an 8-h average. Between 2001 and 2010 the national CO levels ranged between 2.5 and 3.5 ppm on an 8-h average. The current results show that a 15 s sample (i.e., three puffs) of air from the hood, air through the aerosol pump, air through the smoke pump and ECIG-generated aerosol have CO levels ranging between 0.006 and 0.010 μmol/L, well-below the established national average. Furthermore, if the concentration of CO achieved from a 15 s (i.e., 3 puffs) smoke sample is converted to mg/cigarette, the estimated value (≈ 13–20 mg CO/cigarette) is surprisingly close to the range of values (5.9–17.4 mg CO/cigarette) reported by Calfat et al. (Calafat et al., 2004).

Table 5 is assembled from the trace metal contents obtained in Table 4 after accounting for values that are pre-existing on the control MCE membranes and the percent recovery of ECIG-generated aerosol. Furthermore, it lists the estimated contents (μg/cigarette equivalent) of Al, As, Cd, Cu, Fe, Mn, Ni, Pb, and Zn before vaporization of the E-liquid (see Table 3). Although extremely low (2 ng/cig equivalent), to our knowledge, this is the first time the presence of As in ECIG-generated aerosol is reported. The contents of Al, As, Ni, and Zn are all higher in the ECIG-generated aerosol than in the E-liquid before aerosolization, suggesting that the source of these metals is the ECIG device. This is not surprising considering that these metals are used in the construction of the core assembly as indicated in Table 2. For example, the sources of Ni (the only metal captured on MCE membranes exposed to aerosol that is significantly higher than control) are most likely the core tip, the resistance coil and the wiring and welding within the core assembly. It is surprising, however, to find high Al content in the aerosol, especially when the only Al in the core assembly is the woven tube (<10.5%). It is equally surprising to find low Fe content, especially when the content of Fe in the core casing is high (>78%). However, these discrepancies could very well be a function of the solubility of the metal alloy used in the construction of the core assembly, which in turn would affect the metal transfer to aerosol. From this data it appears that there is more Fe in the E-liquid before aerosolization as compared to after aerosolization, but these amounts are so low and so similar that it is unlikely to make any significant difference. Other differences between the values of all metals reported from ECIG-generated aerosol in this study with those reported in the literature are most likely due to methodological variations. It is entirely possible that the presence of these metals pre-existing in the MCE membranes, the inline chamber and the peristaltic pump tubing, along with differences in ECIG construction materials, are responsible for the differences observed when comparing the metal content values of this study with those reported in the literature (Goniewicz et al., 2013b; Williams et al., 2013, 2015; Lerner et al., 2015).

**Table 5:** **Accumulation of trace metals on MCE membranes exposed to ECIG-generated aerosol**.

	Estimated aerosol contents (μg/cig)^*^	**Reported contents in aerosol (μg/cig)**	**References for reported contents in previous column**	**E-liquid (μg/cig equivalent)^@^**	**Primary source of metal**
Al	4.356	0.394	Williams et al., 2013	0.003	ECIG Device
As	0.002	NA	NA	0.000	ECIG Device
Cd	BDL	0.015–0.017	Goniewicz et al., 2013b	BDL	–
Cu	BDL	0.203, BDL–2.03 and 0.365–3.371	Williams et al., 2013, 2015; Lerner et al., 2015	BDL	–
Fe	0.001	0.52	Williams et al., 2013	0.002	E-liquid
Mn	BDL	0.066	Williams et al., 2013	0.000	–
Ni	0.217	0.005 and 0.021–0.029	Goniewicz et al., 2013b; Williams et al., 2013	0.000	ECIG device
Pb	BDL	0.017 and 0.006–0.007	Goniewicz et al., 2013b; Williams et al., 2013	BDL	–
Zn	0.929	0.058 and BDL–0.127	Williams et al., 2013, 2015	0.000	ECIG device

* *Value determined (for 15 puffs on an ECIG or 1 cigarette equivalent) after accounting for the trace metals on control MCE membranes and a 3% recovery (of our vaping system)*.

@ *Values from Table 3*.

*MCE, mixed cellulose ester. BDL, below detection limit or in the event that control MCE membranes have a greater value than the MCE membranes exposed to aerosol. NA, not available*.

The Ni results of this investigation are in agreement with Saffari et al. (2014) who indicate the average concentration of Ni in indoor air after vaping (at a rate of one puff *per* minute for 7 min) is slightly higher than its control outdoor concentration. Williams et al. (2013, 2015) were also able to detect quantities of Ni in ECIG-generated aerosol (ranging from 0 to 50 ng/10 puffs of an ECIG depending on the brand of E-liquid aerosolized), but they do not compare their findings to any control reference, other than to previously published values for Ni in cigarette smoke. On the other hand, Goniewicz et al. (2013b) report Ni to increase between 24 and 71% above the blank sample, although from their methodology it is unclear what constitutes a blank sample. Since the E-liquid used in this study had negligible quantities of Ni, the source of Ni recovered on the MCE membrane exposed to aerosol must be from the ECIG's core assembly. Indeed, elemental analysis reveals that the core, coil, thick wire and weld joint of the core assembly contains much Ni. Furthermore, the core itself appears to be coated with Ag, with the apparent intention to improve electrical conduction. Williams et al. (2015) corroborates these results reporting substantial amounts of Ni, along with Cu, Zn, Ag, and Cr in the core assemblies they analyzed. While the present data indicates no significant differences in the contents of all other trace metals on the MCE membranes exposed to ECIG-generated aerosol compared to control membranes (Table 4), Goniewicz et al. (2013b) reported substantial increases in Cd, and Pb over the blank sample and Lerner et al. (2015) reported a sizeable increase in Cu when compared to their control.

Using the values obtained in Table 4, the estimated contents (μg/cigarette) of Al, As, Cd, Cu, Fe, Mn, Ni, Pb, and Zn in mainstream smoke, after accounting for the pre-existing presence of these trace metals on the control MCE membranes and the percent recovery of cigarette smoke, are listed in Table 6. With the exception of Al and Mn, which are high, all other trace elements in mainstream cigarette smoke are generally comparable to content values reported by others (Schneider and Krivan, 1993; Stohs et al., 1997; Kazi et al., 2009; Mohammad, 2014). At this time it is unclear as to why As in the tobacco and paper is below detection limit, but the possibility exists that As (V), the predominate As species in tobacco (Liu et al., 2012), complexes with silicates (Pappas, 2011), and as such, is not normally dissolved by using the methodology outlined in EPA protocol 3050B (United States Environmental Protection Agency, 1996), thus making it more difficult to detect using ICP-MS. On the other hand, As (III), the predominate As species in smoke condensate and cigarette ash, is more soluble (Liu et al., 2012). Furthermore, the final dilution volume for the tobacco and paper is 200 ml vs. the final dilution volume for MCE membranes which is only 50 ml makes it that much more difficult to detect As in the tobacco. According to Stohs et al. (1997), ~10% of total As appears in mainstream tobacco smoke. Assuming 10% is accurate; this study shows about 0.563 μg of As *per* cigarette, a value that is in line with previously published values (Chiba and Masironi, 1992; Fresquez et al., 2013). Any other discrepancies of trace metal contents in mainstream smoke is most likely due to methodological differences by which the smoke is collected since the trace metal content of tobacco and paper (before combustion) are comparable with those values reported by a number of other investigators (Chiba and Masironi, 1992; Bernhard et al., 2005; Pourkahabbaz and Pourkahabbaz, 2011; Yebpella et al., 2011; Fresquez et al., 2013). All content values of trace metals on smoke exposed MCE membranes are higher than the content values of trace metals on aerosol exposed membranes (as indicated in Table 4), although Cd, Pb, and Zn are not significantly higher. These results are mostly in agreement with Saffari et al. (2014) who reported the indoor concentrations of Cd, Cu, Fe, Mn, Pb, and Zn were all much higher after smoking a cigarette compared to vaping an ECIG, although the indoor concentrations for Al and Ni after smoking or vaping were about the same.

**Table 6:** **Accumulation of trace metals on MCE membranes exposed to conventional cigarette smoke**.

	Estimated smoke contents (μg/cig)^*^	**Reported contents in smoke (μg/cig)**	**References for reported contents in previous column**	**Tobacco (μg/cig)^@^**	**Estimated percent (%) transfer to mainstream smoke**
Al	8	0.342 and 0.22	Stohs et al., 1997; Kazi et al., 2009	240	4
As	0.06	0.0041 and 0.012–0.022	Schneider and Krivan, 1993; Stohs et al., 1997	BDL	–
Cd	0.08	0.065, 1.05, 0.016, and 0.007–0.35	Schneider and Krivan, 1993; Stohs et al., 1997; Kazi et al., 2009; Mohammad, 2014	0.26	31
Cu	0.05	0.013, 0.018 and 0.19	Schneider and Krivan, 1993; Stohs et al., 1997; Mohammad, 2014	4.29	1
Fe	0	0.0168 and 0.42	Schneider and Krivan, 1993; Stohs et al., 1997	243	<0.1
Mn	2	0.0026 and 0.003	Mohammad, 2014; Saffari et al., 2014	72	3
Ni	?	0.00146, 0.632 and 0.0–0.51	Schneider and Krivan, 1993; Stohs et al., 1997; Kazi et al., 2009	1	?
Pb	0.01	0.032, 0.289, 0.094 and 0.017–0.98	Schneider and Krivan, 1993; Stohs et al., 1997; Kazi et al., 2009; Mohammad, 2014	0.53	3
Zn	1	0.127, 0.322 and 0.12 –1.21	Schneider and Krivan, 1993; Stohs et al., 1997; Mohammad, 2014	12	12

* *Value determined (for 15 puffs on a cigarette) after accounting for the trace metals on control MCE membranes and for a 6% recovery (of the smoking system)*.

@ *Values from Table 3*.

*MCE, mixed cellulose ester. ?, Not conclusive since five of eight values from Table 4 are above detection limit. BDL, below detection limit*.

The estimated percent transfers (i.e., from tobacco to mainstream smoke) are also listed in Table 6. Calculated percent transfers of Cd, Ni, and Zn are 31.3, 0.5, and 12.1%, respectively. In comparison, Menden et al. (1972) reported percent transfer to mainstream smoke to be between 7.0 and 10.1% for Cd, between 0.4 and 2.6% for Ni and between 0.4 and 1.5% for Zn. In contrast, Chiba et al. (Chiba and Masironi, 1992) state that 70% of Cd and 70% of Zn in a cigarette are passed on to smoke, but make no distinction between side stream or mainstream smoke. The percent transfers of Cu and Pb from tobacco to mainstream smoke are 1.2% and 2.7%, respectively, and are similar to values obtained from Mohammad et al. (Mohammad, 2014). The percent transfer of Al from tobacco to mainstream smoke is 3.5%, which is high compared to percent transfer determined from Kazi et al. (2009). The reason for this high percent transfer is most likely a reflection of the high Al content in mainstream smoke. Mn content in mainstream smoke is also high when compared to Mn content in smoke reported by others (Schneider and Krivan, 1993; Stohs et al., 1997), but, still, this only accounts for 2.5% transfer of Mn from tobacco to mainstream smoke. The percent transfer of Fe to mainstream smoke is also less than 1%, and is in agreement with Shaikh et al. (2002).

The estimated contents (μg) of Al, As, Cd, Cu, Fe, Mn, Ni, Pb, and Zn from the vaporization of E-liquid equivalent to 20 cigarettes or from the combustion of 20 Marlboro cigarettes are determined from the μg/cigarettes found in Tables 5, 6, respectively, and are listed in Table 7. The recommended exposure limits (REL) published by the National Institute for Occupational Safety and Health (NIOSH) and the permissible exposure limits (PEL) published by the Occupational Safety and Health Administration (OSHA) (United States Department of Labor, 2013) for inhalation of these trace metals are also listed in Table 7. Using the average tidal volume (587 ml) from a 2013 study (Bandyopadhyay et al., 2013) performed on 87 non-smoking male university students (ages 19–24 years) and a respiratory rate of 12 b/min (normal range is 12–16) a total ventilation rate of approximately 7 l/min is achieved. Applying this ventilation rate to either the REL or PEL, an estimate of the maximum allowed inhaled content of each trace metal can be calculated (see Table 7). After comparing the estimated smoke and aerosol contents of all the trace metals following 300 puffs (i.e., 20 cigarettes) with the estimated maximum allowed content for the inhalation of each of these trace metals, Ni inhalation via the ECIG device emerges as the most significant. Vaping the equivalent of a pack of cigarettes can result in 25% of the maximum allowable inhalation of Ni, while the contents of all other trace metals are below 1% of the maximum allowable inhalation. In reality, this level of Ni inhalation is not likely to induce serious health risks in most people, given that RELs and PELs are generally derived using overly cautious principles of safety, nevertheless, Ni is a known potential carcinogen (United States Department of Labor, 2013) and the pathophysiological responses to Ni inhalation is not the same for all individuals. The other two potentially carcinogenic trace metals (Cd and As) (United States Department of Labor, 2013), present more of a concern when smoking. Smoking one pack of cigarettes *per* day can garner up to 10 and 3% of the estimated maximum allowance of Cd and As inhalation, respectively. While these values appear low as compared to maximum allowable inhalation based on OSHA's PEL, a number of studies (Cunningham et al., 2011; Xie et al., 2012; Baumung et al., 2016) utilizing the margin of exposure (MOE) approach (i.e., ratio of the toxicological threshold determined from various data bases to the estimated human intake; where compounds with MOE values less than 10,000 are considered high risk), determine both Cd and As to present considerable health related risks to the consumer; more so for Cd than As. On the other hand, while the maximum allowable inhalation of Ni, based on OSHA's PEL, from ECIG-generated aerosol was higher than the maximum allowable inhalation of Cd and As in mainstream smoke, Xie et al. (2012), using the MOE approach, determined Ni in cigarette smoke to be less concerning than either Cd or As. Determining the likelihood of detrimental pathology occurring from individual constituents of ECIG-generated aerosol using the MOE approach is both intriguing and appealing, particularly for the proponents of harm reduction, since a MOE value could be used as an alternative means of comparing the relative amount of harm associated with “vaping” vs. smoking.

**Table 7:** **Comparison of accumulated trace metals with maximum allowed inhalation**.

	Estimated smoke contents (μg) after 300 puffs^*^ (20 cigarettes) over an 8 h period	Estimated aerosol content (μg) after 300 puffs^*^ (20 cigarettes equivalent) over an 8 h period	**NIOSH REL (μg/L)**	**OSHA PEL (μg/L)**	**Estimated maximum allowed inhalation (μg) based on REL and total ventilation of 7 L/min over an 10 h period**	**Estimated maximum allowed inhalation (μg) based on PEL and total ventilation of 7 L/min over an 8 h period**
Al	170 (1%)	87 (1%)	5.000	5.000	21,000	16,800
As^#^	1.13 (3%)	0.05 (0%)	0.002	0.010	8	34
Cd^#^	2 (10%)	BDL	NA	0.005	NA	17
Cu	1 (0%)	BDL	0.100	0.100	420	336
Fe	0.41 (0%)	0.01 (0%)	5.000	5.000	21,000	16,800
Mn	37 (6%)	BDL	1.000	0.200	4200	672
Ni^#^	?	4 (25%)	0.015	0.500	63	17
Pb	0.3 (0%)	BDL	0.050	0.050	210	168
Zn	29 (0%)	9 (0%)	NA	5.000	NA	16,800

* *Twenty cigarettes equal 300 puffs or one pack of cigarettes*.

# *Potential cancer causing agent*.

*Values in parenthesis indicate the percentages of maximum allowed inhalation based on PEL. MCE, mixed cellulose ester; NIOSH, National Institute for Occupational Safety and Health; REL, recommended exposure limit; OSHA, Occupational Safety and Health Administration; PEL, permissible exposure limit; NA, not available. ?, Not conclusive since five of eight values from Table 4 are above detection limit. BDL, below detection limit. BDL, below detection limit or in the event that control MCE membranes have a greater value than the MCE membranes exposed to aerosol*.

Of course, the high levels of Ni detected in the aerosol of this study is a manifestation of the particular ECIG device/E-liquid combo chosen and does not translate to high Ni content for all device/refill solutions on the market. While others (Goniewicz et al., 2013b; Williams et al., 2013, 2015) have not found levels of Ni in ECIG-generated aerosol to be as high as the levels detected in this investigation, there is indication that the content of metals in aerosol does vary with the device used. This is evident in Williams et al. (2015), who found variations in Sn, Cu and Zn, in addition to Ni, when comparing the aerosols of four different brands of cartridge type ECIG devices (i.e., ECIG devices that are sold complete with a cartridge containing various flavored E-liquids). Due to limitations in their study, Goniewicz et al. (2013b) could not conclude if the ECIGs alone were responsible for the source of Cd, Ni, and Pb in the aerosol of twelve brands of cartridge type ECIGs, but the values they did obtain for Cd, Ni and Pb ranged widely, between 0.01 to 0.22, 0.11 to 0.29, and 0.03 to 0.57 μg, respectively. Another point to be made in defense of the higher levels of Al, Ni, and Zn detected in aerosol of this study, as compared to the aforementioned studies, could be (at least partially) a reflection of the larger core assembly within the plastic tank vs. the smaller size of the cartridge type ECIG devises.

The carcinogenicity of Ni is related to its ability to form nickel carbonyl (Ni(CO)_4_) in the presence of carbon monoxide(Chiba and Masironi, 1992). Very little carbon monoxide is produced by vaping, compared to the massive amounts produced by smoking (see Figure 7). However, the presence of Ni in ECIG-generated aerosol could present an increased risk of carcinogenicity, especially among dual-use individuals (i.e., those individuals who both use ECIGs and smoke). With the advent of new generation temperature controlled (TC) ECIG devices, Ni toxicity becomes an even more critical issue. TC devices do not actually monitor the temperature of coils, but rather the resistance of coils which is then used to calculate coil temperature. The temperature on the TC device is set according to individual preference for vapor production and taste. If the set temperature is exceeded, the ECIG device will shut down. For the user, the advantages of TC are that it prevents dry or burnt puffs, prevents overheating of the device, and prolongs the life of the coil. The problem with TC devices is that they exclusively use coils made of 99% pure Ni. Pure Ni, also referred to as Ni200, is the best material available to construct coils for TC enabled devices. The reasoning is that the resistance of Ni200 coils is extremely low, but increases significantly as the coil heats. Consequently, temperature can be precisely calculated when using coils constructed of Ni200. Kanthaul (an alloy of ferric Fe, Cr and Al), another popular material used to construct ECIG coils, represents the opposite extreme. Kanthaul resistance is extremely high, but changes very little regardless of its temperature. NiChrome (an alloy of 80% Ni and 20% Cr) is another popular material used to construct ECIG coils. It is suspected that the coils used in these ECIG devices are constructed of Nichrome since elemental analysis identified Ni (76%) and Mn (8%) as the major constituents. Although no Cr was detected in the coil *per se*, elemental analysis at the juncture of thick extension wire, coil and weld joint revealed 53% Ni and 18% Cr (see Table 2). It is possible that Cr is misidentified as Mn, since their X-ray energies at Kα_1_, Kβ_1_, Lα_1_, and Lβ_1_, are fairly close (Periodic Table of Elements and X-rays Energies, 2015) (e.g., 5.900 KeV for Mn and 5.415 KeV for Cr at Kα_1_). Other potential misidentifications include Zn as gallium and Sn as antimony for (see Table 2). The presence of Ni in many commercially available ECIG devices, coupled with its presence in ECIG-generated aerosol, could potentially lead to health related issues such as reactions induced by Ni allergies or even cancer. Consequently, the use of excessive Ni in the manufacturing of ECIG devices should be minimized.

The pathophysiological effects of the other trace metals in cigarette smoke have previously been reviewed (Chiba and Masironi, 1992; Bernhard et al., 2005) and it is not the intent of this report to delve further into the matter. However, in light of the finding concerning the high levels of Al reported in Tables 5, 6 for both ECIG-generated aerosol (4 μg/cig equivalent) and cigarette smoke (8 μg/cig), it is necessary to mention the fact that Al accumulation in neural tissue may be correlated with Alzheimer's disease (Tomljenovic, 2011). It is evident that Al is abundant in the construction of many ECIG devices. Williams et al. (2013) list Al as the fifth most concentrated element of the 21 they analyzed in ECIG-generated aerosol. From the low levels of all other trace metals shown in Table 5 and their relationship to estimated maximum allowed inhalation shown in Table 7, it is unlikely that the other trace metals detected in ECIG-generated aerosol pose any serious pathological risks.

Although Si and Sn recovered from MCE membranes exposed to either ECIG-generated aerosol or cigarette smoke were not measured in the present investigation, elemental analysis of the core assembly identified Si as a major element of the upper core cover, gasket, fabric material, woven tube, and wick fibers and a small amount Sn (6%) on the inner side of the woven tube. These results are somewhat in agreement with Williams et al. (2013, 2015). In one study (Williams et al., 2013) they identified Si (2.24 μg/10 puffs) to be among the top three elements with the highest aerosol concentrations; only sodium (4.18 μg/10 puffs) and boron (3.83 μg/10 puffs) had higher aerosol concentrations than Si. In another study (Williams et al., 2015), they found Sn to be concentrated in the weld joints of only one brand of ECIG device and the amount of Sn in the aerosol of this brand was about 4 μg/10 puffs. All other brands of ECIGs they tested had very little Sn in their makeup and was reflected as such in the generated aerosol.

This investigation undertakes an important subject concerning the presence of trace metals in ECIG-generated aerosol, but there are limitations to this study. While levels of Ni were detected in the aerosol that substantially exceed control levels in the single ECIG device/E-liquid used, it cannot be assumed that this is the case for all ECIG device/E-liquid combinations. What it does convey, however, is the existent of a possibility that other ECIG devices available on the market may also transfer Ni from the device to the aerosol, especially for those devices that use NiChrome or Ni200 resistance coils. The fact that five of the eight Ni samples trapped on MCE membranes exposed to smoke exceeded the upper limit of IPC-MS instrumentation is another limitation. Consequently, a statement regarding differences between the levels of Ni on the MCE membranes exposed to aerosol and the levels of Ni on MCE membranes exposed to smoke cannot be made. On the other hand, it can be stipulated that the E-liquid used is not responsible for this Ni transfer since the level of all trace metals analyzed in the E-liquid were extremely low and no other studies, to our knowledge, show any different. Hess et al. (2017) did find high concentrations of trace metal (specifically Cd, Cr, Pb, Mn, and Ni) in the E-liquid of five brands of ECIG cartridges, but this is not the same as E-liquid that has never touched an ECIG device. Thus, the variation in the levels of trace metals they reported could well be due to the brand of ECIG cartridges they tested and not the E-liquid *per se*. Another limitation of the present study relates to the possibility of silicates binding As in tobacco and could well be the reason As levels in tobacco were undetectable (Johnson et al., 2006; Liu et al., 2012). In retrospect, an alternative means of digesting As, such as a microwave digestion process followed by ICP-MS (Fresquez et al., 2013) may have been a better choice. The determination of As in cigarette smoke or ECIG-generated aerosol presents another interesting problem concerning its speciation since As III, the primary species found in smoke, is more toxic to humans than As V, the primary species found in tobacco (Heikens et al., 2007; Pappas, 2011). While levels of As in E-Liquid were extremely low, its speciation in E-liquid or ECIG-generated aerosol is not clear since ICP-MS cannot distinguish between the two As species. Identification of which As species is present in E-liquid and ECIG-generated aerosol is thus critical in comparing As toxicity induced by vaping to that of smoking. On the other hand, the amount of As detected on MCE membranes exposed to aerosol was significantly less than what was found on membranes exposed to smoke and not different from background control. Consequently, it is unlikely that As generated from the ECIG-Device/E-liquid combination used in this investigation is a significant cause of concern.

In summary, a smoke/aerosol system which can be used to effectively measure trace metals (as well as other low concentration compounds) in conventional cigarette smoke or ECIG-generated aerosol has been established. Currently this system is being used to investigate the absence or presence of nicotine, nicotine related alkaloids and tobacco specific nitrosamines in both ECIG-generated aerosol from a number of commercially available E-liquids and cigarette smoke. It is worth mentioning that in an effort to improve the percent recovery of nicotine, the surface area on which aerosol/smoke is collected has been increased by switching from a 13 mm to a 25 mm membrane. In general, the findings of this study suggest that the concentrations of most trace metals extracted from cigarette smoke exceed the concentrations of trace metals extracted from ECIG-generated aerosol. While confident of these findings, it must be emphasized that these results are specific to the single ECIG device/E-liquid combination used. Nevertheless, a possibility for significant trace metal inhalation exists depending on the brand of ECIG device used. The present study illustrates this point. Given that Ni in the E-liquid is nearly undetectable, the source of Ni in the aerosol must be the ECIG device. From this study, it is unlikely that the ECIG-generated aerosol contains enough of the other trace metals to induce significant pathology.

## Author contributions

DP: Deviced puffing protocol and developed experimental design, had primary oversight of all experiments, and wrote the manuscript with the editorial assistance of the other authors. AC: Performed the validation experiments (i.e., inlet out temperature, MCE composition, etc.) and experiments involved with trapping of trace metals on MCE membranes. JN: Performed collected the elemental analysis data of the core assembly using EDS. RJ: Collected and analyzed the carbon monoxide data and donated laboratory equipment essential to the completion of this study.

### Conflict of interest statement

The authors declare that the research was conducted in the absence of any commercial or financial relationships that could be construed as a potential conflict of interest.
